# Altered resting-state EEG source functional connectivity in schizophrenia: the effect of illness duration

**DOI:** 10.3389/fnhum.2015.00234

**Published:** 2015-05-05

**Authors:** Giorgio Di Lorenzo, Andrea Daverio, Fabiola Ferrentino, Emiliano Santarnecchi, Fabio Ciabattini, Leonardo Monaco, Giulia Lisi, Ylenia Barone, Cherubino Di Lorenzo, Cinzia Niolu, Stefano Seri, Alberto Siracusano

**Affiliations:** ^1^Laboratory of Psychophysiology, Chair of Psychiatry, Department of Systems Medicine, University of Rome “Tor Vergata”Rome, Italy; ^2^Chair of Psychiatry, Department of Systems Medicine, University of Rome “Tor Vergata”Rome, Italy; ^3^Psychiatric Clinic, Fondazione Policlinico “Tor Vergata”Rome, Italy; ^4^Department of Medicine, Surgery and Neuroscience, University of SienaSiena, Italy; ^5^Berenson-Allen Center for Non-Invasive Brain Stimulation, Beth Israel Medical Center, Harvard Medical SchoolBoston, MA, USA; ^6^Don Carlo Gnocchi Onlus FoundationMilano, Italy; ^7^School of Life and Health Sciences, Aston Brain Centre, Aston UniversityBirmingham, UK

**Keywords:** schizophrenia, psychosis, brain oscillations, disconnectivity, synchronization, excitatory/inhibitory dysfunction, neural plasticity, brain network

## Abstract

Despite the increasing body of evidence supporting the hypothesis of schizophrenia as a disconnection syndrome, studies of resting-state EEG Source Functional Connectivity (EEG-SFC) in people affected by schizophrenia are sparse. The aim of the present study was to investigate resting-state EEG-SFC in 77 stable, medicated patients with schizophrenia (SCZ) compared to 78 healthy volunteers (HV). In order to study the effect of illness duration, SCZ were divided in those with a short duration of disease (SDD; *n* = 25) and those with a long duration of disease (LDD; *n* = 52). Resting-state EEG recordings in eyes closed condition were analyzed and lagged phase synchronization (LPS) indices were calculated for each ROI pair in the source-space EEG data. In delta and theta bands, SCZ had greater EEG-SFC than HV; a higher theta band connectivity in frontal regions was observed in LDD compared with SDD. In the alpha band, SCZ showed lower frontal EEG-SFC compared with HV whereas no differences were found between LDD and SDD. In the beta1 band, SCZ had greater EEG-SFC compared with HVs and in the beta2 band, LDD presented lower frontal and parieto-temporal EEG-SFC compared with HV. In the gamma band, SDD had greater connectivity values compared with LDD and HV. This study suggests that resting state brain network connectivity is abnormally organized in schizophrenia, with different patterns for the different EEG frequency components and that EEG can be a powerful tool to further elucidate the complexity of such disordered connectivity.

## Introduction

Disordered brain connectivity at cortical level, generally defined as failure of effective functional integration within and between brain areas, has been proposed as a *core* deficit of schizophrenia. This conclusion is based on neuroimaging evidence on structural, functional, and effective brain connectivity (Friston, 1998; Ribolsi et al., 2009; Schmitt et al., 2011). Structural Connectivity (SC) refers to the anatomical substrate of defined fiber pathways connecting different brain regions (Koch et al., 2002), whereas Functional Connectivity (FC) is defined as the temporal correlation of the activities of different cortical/brain regions (Fingelkurts et al., 2005). The definition of Effective Connectivity (EC) has been more contentious (Horwitz, 2003), referring to direct or indirect influence that one neural system exerts over another (Friston, 2011).

FC impairment in schizophrenia has been extensively investigated. Studies using functional Magnetic Resonance Imaging (fMRI) have shown abnormal FC both in first episode/early stage (Begre and Koenig, 2008) and in chronic schizophrenic patients (Lynall et al., 2010; Fitzsimmons et al., 2013; Wang et al., 2014). EEG studies, capitalizing on its high temporal resolution, have shown impaired FC in all frequencies bands of the EEG spectrum (Stephan et al., 2006). Transcranial magnetic stimulation (see for review Rogasch et al., 2014) and transcranial direct current stimulation (Hasan et al., 2013; Ribolsi et al., 2013) studies have identified altered inhibitory/excitatory properties of brain networks and of interhemispheric connectivity in schizophrenia. Finally, confirmation of the central role of disordered connectivity has also emerged from animal models of schizophrenia (Dickerson et al., 2010; Gruber et al., 2010).

In schizophrenic patients, altered FC in response to cognitive and sensory paradigms has been demonstrated in all the traditional EEG frequency bands (Pachou et al., 2008; Fujimoto et al., 2013) supporting the hypothesis that electroencephalographic indices of FC may be a useful marker of specific impairment in higher-order processing (see the review of Uhlhaas et al., 2008). Furthermore, EEG-FC in the resting state reflects the activity of interneuron connections and cortical synchronization that are temporally interrupted during specific tasks (Cabral et al., 2014) and has the potential to reveal an *a priori* intrinsic dysfunctionality which may represent either a state or trait-marker of the schizophrenic condition.

An important limiting factor in some EEG-FC studies is that the majority of connectivity measures are based on scalp sensors, which may cause spurious connectivity patterns due to the vulnerability of EEG signal to volume conduction phenomena (Nolte et al., 2004). To address this issue, a number of electrical source imaging methods have been proposed as a valid alternative to channel-related FC (Thatcher et al., 2007; Schoffelen and Gross, 2009; Sakkalis, 2011). In this context, an index of physiological “lagged connectivity” between pairs of brain sources capable of minimizing non-cerebral artifacts and that is not affected by active reference electrodes was recently proposed (Pascual-Marqui et al., 2011). This method has been successfully applied to investigate EEG Source Functional Connectivity (EEG-SFC) between cortical regions in health (Stein et al., 2013) and disease (Canuet et al., 2011, 2012; Clemens et al., 2011; Pagani et al., 2012; Olbrich et al., 2014).

Using this approach, Lehmann and colleagues showed aberrant resting-state EEG cortical connectivity pattern in first episode, drug-naïve schizophrenic patients (Lehmann et al., 2014).

The primary aim of this study was to investigate differences in resting-state EEG SFC between a group of clinically stable, medicated schizophrenic patients, and healthy controls. A secondary aim was to investigate the effect of disease duration on source connectivity patterns in the group of schizophrenic patients.

## Materials and methods

### Subjects

From November 2007 to October 2012, patients between 18 and 65 years of age from the outpatient program of “Tor Vergata” University Psychiatry Clinic with a diagnosis of schizophrenia (SCZ) were considered for the study. The inclusion criterion was a stable clinical and pharmacological condition from at least 3 months. The exclusion criteria were history of head trauma, mental retardation, and history in the last 6 months of alcohol, and/or substance abuse. In order to study the effect of illness duration patients were divided in those with short disease duration (SDD, with less than 5 years from the first psychotic episode) and those with long disease duration (LDD, more than 5 years from the first psychotic episode). During the same period, healthy volunteers (HV) of comparable age and socio-economical status without a personal and familiar history of psychiatric disorders were enrolled as controls. The MINI-Plus (Sheehan et al., 1998) was used to confirm the diagnosis of SCZ according to DSM-IV criteria in the clinical sample and the absence of psychiatric diagnosis in the control group. The Positive and Negative Syndrome Scale (Kay et al., 1987) was used to assess the severity of psychopathology. Based on the recent literature (Van den Oord et al., 2006; van der Gaag et al., 2006; Wallwork et al., 2012; Liemburg et al., 2013) the 30 items (7 for Positive symptoms, 7 for Negative symptoms and 16 for General psychopathology) were rearranged in six dimensions: Positive (POS; P1 + P3 + P5 + P6 + G9); Negative – Expressive (NEG-EXP; the core negative symptoms characterized by expressive deficits; N1 + N3 + N6 + G7); Negative - Social (NEG-SOC; social emotive withdrawal/social amotivation; N2 + N4 + G16); Disorganization (DIS; disorganized/concrete/cognitive items; P2 + N7 + N5 + G10 + G11 + G12 + G5 + G13); Excitement (EXC; P4 + P7 + G8 + G14); Emotional Distress (EMO-D; anxiety and depression; G1 + G2 + G3 + G4 + G6 + G15). The total score of the Calgary Depression Scale for Schizophrenics (CDSS; Addington et al., 1990) was used to measure the depression severity in SCZ sample, independently from confounding factor as the positive and negative symptoms of SCZ and any drug effects. The premorbid Intelligence Quotient (IQ) was investigated, in the HV and SCZ, through a reading task, the *Test di Intelligenza Breve* (TIB; Sartori et al., 1997), an Italian adaptation of the National Adult Reading Test (NART; Nelson, 1982). The Cognition Rating Scale (SCoRS; Keefe et al., 2006; Vita et al., 2013) was used, in the HV and SCZ, as a screening measure of cognitive function. The severity of functional disability was assessed in SCZ with the Social and Occupational Functioning Assessment Scale (SOFAS; American Psychiatric Association, 2000). All subjects enrolled in the study were right-handed on the basis of the Edinburgh Handedness Inventory (Oldfield, 1971). All participants provided written informed consent. The study was approved by the “Tor Vergata” University Hospital ethics committee and carried out in accordance with “Ethical Principles for Medical Research Involving Human Subjects” (Declaration of Helsinki, 1964).

### EEG recordings and data acquisition

The EEG was recorded from 37 scalp locations using a pre-cabled electrode cap (Bionen, Florence, Italy), with Ag/AgCl disk electrodes located at the following positions: FP1, FPz, FP2, AF7, AF3, AF4, AF8, F7, F3, Fz, F4, F8, CP5, CP1, CP2, CP6, T7/T3, C3, Cz, C4, T8/T4, CP5, CP1, CP2, CP6, P7/T5, P3, Pz, P4, P8/T6, PO7, PO3, PO4, PO8, O1, Oz, O2. Recording channels were connected to a reference electrode located in the AFz position and the ground electrode was in POz. Electrode impedances were kept to less than 10 KΩ. The signal was amplified by 40-channel EEG device (Galileo MIZAR-sirius, EBNeuro, Florence, Italy) and acquired with GalNT software. Data were collected with a sampling rate (SR) of 1024, hardware EEG High-Pass filter at 0.099 Hz and Low-Pass at 0.45/SR (0.45 × 1024 Hz = 460.8 Hz).

Participants were instructed not to drink coffee, tea, or any other beverage containing stimulants and to refrain from smoking cigarettes in the 2 h before the beginning of the recording session. Information on the quality of sleep during the night prior to the recording was collected and the EEG session was postponed if the subject reported a non-restoring sleep. Women were recorded during the follicular phase of menstrual cycle. EEG was continuously recorded for 3 min while the subjects were at rest with closed eyes. Participants sat comfortably in an armchair, in a sound attenuated room and were instructed to relax while staying awake with eyes closed and to limit eye movements.

### EEG preprocessing

EEG data was exported to EDF from the native format using NPX Lab 2012 (publicly available software at www.brainterface.com) and analyzed in the EEGLAB environment (http://sccn.ucsd.edu/eeglab/index.html; Delorme and Makeig, 2004), a collection of analytical tools running under Matlab 7.7.0 R2010a (Mathworks Inc., Natick, MA). EEG signal was digitally band-pass filtered between 1 and 100 Hz (with a FIR filter) and re-referenced to the average reference. After visual inspection and manual removal of segments characterized by gross artifacts, non-cerebral source activities (eye blinks and movements, cardiac and electromyographic activity) were identified and rejected using a semiautomatic procedure (Medaglia et al., 2009; Porcaro et al., 2009). The EEG signal was first decomposed into independent components (ICs) using FastICA version 2.5 (Hyvarinen and Oja, 2000; http://www.cis.hut.fi/projects/ica/fastica). ICs corresponding to artifactual sources and brain activity were separated with a manual procedure (Medaglia et al., 2009; Porcaro et al., 2009). The electrical power line noise was removed on ICs using the CleanLine plug-in of EEGLab. After removal of artifactual non-cerebral ICs, the “cleaned” signal was reconstructed by retro-projecting only the ICs containing cerebral signal. Cleaned data were segmented in 2-s epochs for following analysis steps.

### EEG source functional connectivity analysis

This stage was performed using the exact Low Resolution Brain Electromagnetic Tomography (eLORETA) software (available at http://www.uzh.ch/keyinst/loreta.htm) and consisted of two steps

1. Region of interests (ROI), to identify intracerebral electrical sources;
2. EEG-SFC to compute the functional connections between the identified sources.

#### ROIs

The current implementation of eLORETA (Pascual-Marqui, 2007) uses a realistic head model (Fuchs et al., 2002) based on the MNI152 template, with the three-dimensional solution space restricted to the cortical gray matter and hippocampi, as determined by the probabilistic Talairach atlas (Lancaster et al., 2000). The intracerebral volume (eLORETA inverse solution space) is partitioned in 6239 voxels at 5 mm spatial resolution (i.e., cubic elements of 5 × 5× 5 mm). Anatomical labels corresponding to Brodmann areas are reported using neuroanatomical Montreal Neurological Institute space (MNI; Montreal, Quebec, Canada), converted to Talairach space (Brett et al., 2002). In order to identify intracerebral electrical sources and reduce the number of estimates, we chose a ROI-maker method (available in eLORETA) for the construction of the ROIs. Firstly, we selected 28 ROIs (14 for each hemisphere; see Supplementary Table 1), starting from 42 Brodmann Areas (BAs) in each hemisphere according to the anatomical definitions of BAs provided by eLORETA software package, based on the Talairach Daemon (http://www.talairach.org/). Secondly, we selected a single voxel as the centroid of each ROI, as this representative voxel is the closest to the center of the ROI mass (see Supplementary Figure 1, for the complete ROI list and coordinates of each voxel). The reduction to a single ROI voxel was done to avoid potential bias in the analysis due, mainly, to the high correlation among neighboring voxels generated by the relatively low spatial resolution and inherent smoothness of the eLORETA inverse solution.

#### EEG-SFC

We computed the spectral time series of the centroid voxel of each ROI and considered it representative of the spectral activity of the whole ROI. The resulting 378 pairs of intra-cerebral electrical sources were used to estimate FC in the brain (Schoffelen and Gross, 2009). We adopted the eLORETA connectivity algorithm, described in two methodological reports (Pascual-Marqui, 2007; Pascual-Marqui et al., 2011) and applied in recent EEG (Canuet et al., 2011, 2012; Lehmann et al., 2012, 2014; Pagani et al., 2012; Olbrich et al., 2014) and ERP (Mulert et al., 2011) studies. This FC method represents connectivity indices in instantaneous and lagged components. While the instantaneous (zero-lag connectivity) component in a given frequency band is sensitive to intrinsic limitations such as the effect of volume conduction and to low spatial resolution, the lagged (non-instantaneous connectivity) component has a physiological origin. The physiological lagged connectivity index, namely Lagged Phase Synchronization (LPS), measures the functional connection (a corrected lagged phase synchrony value after the instantaneous zero-lag contribution has been excluded) between two intracerebral electrical source signals in the frequency domain based on normalized discrete Fourier transforms. For each ROI pair, we calculated LPS indexes for the following six frequency bands: delta (1.5–4), theta (4–8 Hz), alpha (8–12 Hz), beta1 (12–20 Hz), beta2 (20–30 Hz), and gamma (30–80 Hz).

Four contrasts were analyzed by eLORETA Log-F ratio statistics (with 5000 randomizations): HV vs. SCZ; LDD vs. SDD; HV vs. SDD; HV vs. LDD. In each contrast, a unique Bonferroni-corrected two-tailed *p*-value was produced for all ROI pairs in the six frequency bands (*n* = 2268). Significant threshold was set at *p* < 0.01. The visualization of brain networks was performed using BrainNet Viewer (http://www.nitrc.org/projects/bnv/) Matlab toolbox (Xia et al., 2013).

## Results

Seventy-seven (25 SDD and 52 LDD) SCZ patients and 78 HV were included in the study. Descriptive and univariate statistics of socio-demographic and clinical characteristics of the sample are summarized in Table 1. HV and SCZ did not differ for gender, age, education, and premorbid IQ scales. As expected SCoRS was higher in SCZ, indicating an impairment in general cognitive functioning. No significant differences were found between SDD and LDD other than for age (younger age of the SDD group) and illness duration (longer by definition for the LDD group).

**Table 1:** **Descriptive and univariate statistics of sociodemographic and clinical characteristics of sample**.

	**HV (*n* = 78)**	**SCZ (*n* = 77)**	**SDD (*n* = 25)**	**LDD (*n* = 52)**	**HV vs. SCZ**	**(HV vs.) SDD vs. LDD**
Gender (w/m)	36/42	26/51	11/14	15/37	χ^2^_(1)_ = 2.48, *p* = 0.12	χ^2^_(2)_ = 4.09, *p* = 0.13
Age	32.78 (10.94)	35.44 (11.05)	25.72 (4.32)	40.12 (10.23)	*t*_(153)_ = −1.51, *p* = 0.13	*F*_(2,152)_ = 19.11, *p* < 0.0001 ^a^
Education (years)	13.60 (3.57)	12.75 (3.32)	12.64 (3.16)	12.81 (3.42)	*t*_(153)_ = 1.53, *p* = 0.13	*F*_(2,152)_ = 1.19, *p* = 0.31
Verbal-premorbid IQ	120.73 (8.85)	119.18 (8.85)	118.02 (9.92)	119.74 (8.33)	*t*_(153)_ = 1.09, *p* = 0.28	*F*_(2, 152)_ = 0.91, *p* = 0.40
Performance-premorbid IQ	110.64 (3.75)	110.08 (3.45)	109.74 (3.90)	110.25 (3.23)	*t*_(153)_ = 0.96, *p* = 0.34	*F*_(2,152)_ = 0.60, *p* = 0.54
Total-premorbid IQ	114.76 (4.86)	113.95 (4.68)	113.00 (4.47)	114.41 (4.75)	*t*_(153)_ = 1.06, *p* = 0.29	*F*_(2, 152)_ = 1.30, *p* = 0.28
Illness onset (years)		21.13 (4.82)	22.28 (3.93)	20.58 (5.14)		*t*_(75)_ = 1.46, *p* = 0.15
Illness duration (years)		14.31 (9.90)	3.44 (1.04)	19.54 (7.74)		*t*_(75)_ = −10.32, *p* < 0.0001
Hospitalization		2.95 (1.34)	2.56 (1.42)	3.14 (1.27)		*t*_(75)_ = −1.79, *p* = 0.08
PANSS POS		11.70 (5.93)	10.20 (4.28)	12.42 (6.49)		*t*_(75)_ = −1.56, *p* = 0.12
PANSS NEG-EXP		10.70 (3.65)	11.64 (3.21)	10.25 (3.79)		*t*_(75)_ = 1.58, *p* = 0.12
PANSS NEG-SOC		11.78 (4.22)	12.00 (4.72)	11.67 (2.47)		*t*_(75)_ = 0.32, *p* = 0.75
PANSS DIS		15.31 (6.00)	13.96 (5.05)	15.96 (6.35)		*t*_(75)_ = −1.38, *p* = 0.17
PANSS EXC		6.77 (2.32)	6.64 (2.45)	6.83 (2.28)		*t*_(75)_ = −0.33, *p* = 0.74
PANSS EMO-D		15.40 (6.29)	15.36 (6.04)	15.42 (6.46)		*t*_(75)_ = −0.04, *p* = 0.97
CDSS		4.96 (4.21)	5.84 (3.94)	4.54 (4.31)		*t*_(75)_ = 1.28, *p* = 0.21
Chlorpromazine equivalent		306.10 (167.27)	330.84 (162.44)	294.21 (169.80)		*t*_(75)_ = 0.90, *p* = 0.37
Antipsychotics (SGA/FGA)^b^		65/21	19/6	37/15		
Benzodiazepines (no/yes)		59/18	19/6	40/12		
Antidepressants (no/yes)		59/18	20/5	39/13		
Anticonvulsant (no/yes)		67/10	21/4	46/6		
Anticolinergic (no/yes)		70/7	23/2	47/5		
SCoRS	22.56 (1.73)	33.55 (6.13)	32.24 (5.53)	34.17 (6.36)	*t*_(153)_ = −15.21, *p* < 0.0001	*F*_(2, 152)_ = 118.90, *p* < 0.0001^c^
SOFAS		55.61 (15.85)	58.88 (12.60)	54.04 (17.09)		*t*_(75)_ = 1.26, *p* = 0.21

*HV, Healthy Volunteers; SCZ, people affected by schizophrenia; LDD, people affected by schizophrenia with Long Duration of Disease; SDD, people affected by schizophrenia with Short Duration of Disease. Data are frequencies and means (SDs)*.

*^a^ Bonferroni post-hoc: HV vs. SDD, p = 0.007; HV vs. LDD, p < 0.0002; SDD vs. LDD, p < 0.0001*.

*^b^ In 15 SCZ (6 SDD and 9 LDD; SDD vs. LDD: χ^2^_1_ = *0.48*, p = 0.49) the SGA therapy was associated to low doses of FGA*.

*^c^ Bonferroni post-hoc: HV vs. SDD, p < 0.0001; HV vs. LDD, p < 0.0001; SDD vs. LDD, p = 0.23*.

### EEG-SFC

**Figure 1:**
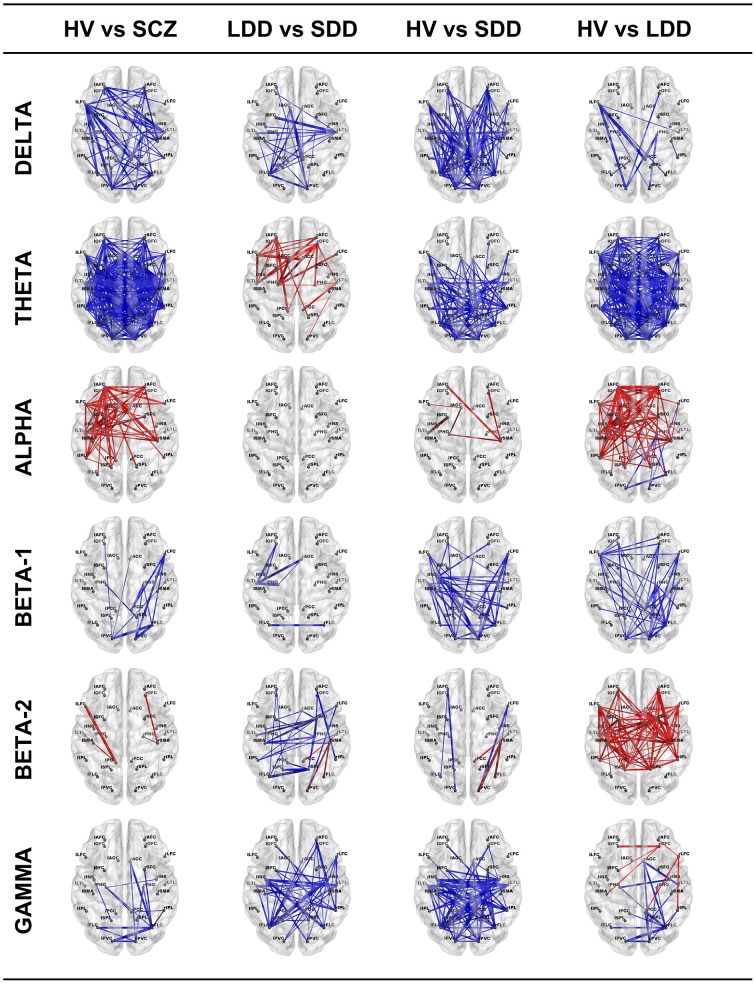
**Resting-state EEG SFC results of the four comparisons in the six bands**. The red color indicates an increase of LPS (functional connectivity) indices and the blue a decrease. The thicker is the line the bigger is the difference. HV, Healthy Volunteers; SCZ, people affected by schizophrenia; LDD, people affected by schizophrenia with Long Duration of Disease; SDD, people affected by schizophrenia with Short Duration of Disease.

Results of lagged non-linear connectivity differences between HV and SCZ, and within the SCZ group between LDD and SDD in all frequency bands are summarized in Figure 1. Tables with detailed results of all comparisons are available as Supplementary Material.

#### Delta band

SCZ had significantly higher connectivity than HV; differences where more evident between left prefrontal and right parieto-temporal ROIs (e.g., lLFC-rLTL), left prefrontal and cingulate ROIs (e.g., lLFC-rPCC) and left prefrontal and occipital ROIs (e.g, lLFC-rFLC, lLFC-lPVC). Patients in the SDD group showed higher connectivity values than those in the LDD group and than HV.

#### Theta band

SCZ showed a widespread higher connectivity between most of ROI pairs compared to HV. Similarly, LDD patients showed diffusely higher connectivity compared with HV. Compared with SDD, LDD had higher connectivity in prefrontal, cingulate, and parieto-temporal ROI pairs (e.g., rLFC-lPHG, lAFC-lPCC). SDD, compared with HV, showed higher connectivity in parieto-temporal, cingulate and occipital ROI pairs with fewer differences in connectivity values of prefrontal ROIs (e.g., lAFC, rAFC, lOFC).

#### Alpha band

SCZ had significantly lower connectivity in prefrontal, parieto-temporal, and cingulate ROI pairs compared to HV. No significant differences were found between LDD and SDD. Decreased connectivity was most evident in LDD compared with HV.

#### Beta1 band

SCZ had significant higher connectivity between occipital, parietal, and prefrontal ROIs compared with HV. In the comparisons between HV and LDD and HV and SDD, both LDD and SDD showed diffusely higher connectivity in respect of HV.

#### Beta2 band

Differences between SCZ and HV in a limited number of ROI pairs (lLFC-lPCC, rOFC-rSMA). SDD had a complex pattern characterized by both increased and reduced connectivity between different ROIs compared with LDD and HV. LDD showed a diffuse reduced connectivity compared with HV.

#### Gamma band

SCZ had significantly higher connectivity between right occipital-right prefrontal, right occipital-right parieto-temporal, and right occipital-right cingulate ROI pairs (rFLC-rSFC,rFLC-rACC, rFLC-rPHG) compared to HV. SDD had significant diffuse increased connectivity compared with HV and LDD. LDD showed a decreased connectivity between prefrontal and parieto-temporal ROI pairs (e.g., rLFC-rIPL) and increased connectivity between occipital and right cingulate ROIs (rFLC-rACC) and between occipital and right temporal ROIs (e.g., rFLC-rPHG) compared with HV.

## Discussion

The results of our study, capitalizing on the additive dimension offered by the functional specificity of the frequency content of EEG, extend prior studies and suggest that brain networks in the resting state are abnormally organized in schizophrenia. The main findings can be summarized as:

- In low frequencies (delta, theta), the SCZ group showed greater EEG-SFC (in particular a widespread increase in theta band) compared to HV and increased frontal connectivity in LDD in theta band compared with SDD.
- In the alpha band, the SCZ group showed greater frontal EEG-SFC compared with HV; no differences were found between LDD and SDD.
- In the beta1 band, the SCZ group presented greater EEG-SFC compared with HV, and in SDD compared with LDD who had the lowest EEG-SFC.
- In beta2 band, the LDD group presented lower frontal and parieto-temporal EEG-SFC compared with HV and with the SDD group.
- In the gamma band, SCZ had lower occipital and cingulate connectivity compared with HV, while SDD had greater connectivity values compared with LDD and HV.

This was one of the first studies to examine EEG-SFC in the resting state and therefore a direct comparison with other studies is not possible. Most of FC EEG studies focused on task-related (cognitive, visual, auditory stimulation) alterations in schizophrenia (see for review Uhlhaas and Singer, 2010). Based on our findings, we can attempt to present a coherent model.

### Hyperconnectivity in low frequencies

An increase in low-frequency oscillations (power) in delta and theta bands at rest is one of the most consistent findings in schizophrenia (Moran and Hong, 2011). Less is known about resting state FC in the segment of the EEG spectrum; earlier studies (see the review of Leocani and Comi, 1999) have found increased coherence in low frequencies (Nagase et al., 1992; Mann et al., 1997). Low-frequency power but not coherence can be modified by pharmacological treatment (Merrin et al., 1989), suggesting that impaired low frequency coherence could be the expression of abnormal cortical organization. In first episode, drug-naïve SCZ, a delta band increase was recently shown in EEG-SFC (Lehmann et al., 2014). Increased resting-state theta band connectivity between fronto-temporo-parietal source-pairs is reported in first episode medicated SCZ, with an effect of mediation on the verbal memory performance. Moreover, recent evidence of abnormal theta connectivity in subjects at high-risk for psychosis has been reported suggesting that impairment in this frequency band could be a trait of the SCZ/psychosis spectrum (Andreou et al., 2015). Our results are largely in line with these findings. Moreover diffuse hyperconnectivity characterizes both early stage (SDD) and chronic (LDD) disease even if only the latter is characterized by a prefrontal involvement. Animal studies have demonstrated that GABAergic inhibition and disinhibition can directly modulate cortical synchrony especially in low frequencies (Xiao et al., 2012); GABA neurotransmission is altered in prefrontal cortex (Volk and Lewis, 2002) and its impairment plays a key role in schizophrenic disease (Stan and Lewis, 2012; Schmidt and Mirnics, 2015). Bearing in mind the limitations in translating data from animal models, increased EEG-SFC at rest in low frequencies may be explained as a result of inefficient modulation (impaired inhibition), leading to aberrant synchrony.

### Frontal alpha hypoconnectivity

As noted above, reduced frontal alpha EEG-SFC is a robust result of our study, independent of the duration of schizophrenic disease course and may represent a valid trait-marker of schizophrenic disease. This finding extends and confirms previous reports on schizophrenic patients (Jetha et al., 2009) and patients with mood disorders (Allen et al., 2004; Vuga et al., 2006). Resting-state EEG deficit in alpha power has been widely reported in schizophrenic patients (see review Boutros et al., 2008) with no difference between first episode and chronic patients (Sponheim et al., 1994). Likewise, reduction of EEG alpha coherence, which is an index of synchronization and connectivity, was noted and related to psychopathological dimension of schizophrenia (John et al., 2002). A recent study investigated resting state alpha band EEG FC in the sensor space and concluded that schizophrenic patients had aberrant anterior alpha FC, more pronounced in the left hemisphere (Peng et al., 2013). Finally, an alpha band decrease from resting-state EEG-SFC was recently shown in first episode, drug-naïve SCZ (Lehmann et al., 2014). Due to the key role of alpha oscillations as neural substrates of attention modulation through the suppression/inhibition and selection functions (Klimesch, 2012), impairment in alpha resting-state connectivity might be one of the main indices of long lasting and stable cognitive deficit observed in SCZ.

### Beta band dysfunction

The increased beta1 EEG-SFC in the SCZ group is mainly seen in the posterior regions of the right hemisphere; SDD showed greater EEG-SFC specifically between left temporal, left orbitofrontal, and right anterior cingulate ROI pairs. When compared separately against HV, LDD, and SDD showed different patterns of increased EEG-SFC. An increase of beta1 EEG-SFC was recently described in patients with at-risk mental state (ARMS) for psychosis, suggesting that atypical beta-band source synchronization could be an indicator of an increased vulnerability for SCZ/psychosis spectrum (Ramyead et al., 2014). Furthermore, patients with short disease duration showed greater EEG-SFC in the beta2 band compared with those with longer duration who show widespread reduction in EEG-SFC compared with HV. Although results of beta2 FC do not appear as univocal as those of the other frequency bands, they could be explained by hypothesizing that the initial pattern of hyperconnectivity, which has indeed been demonstrated in paradigm-related MEG connectivity studies (Sun et al., 2013), could in time be followed by the diffuse deficit seen in chronic patients. Comparison with other EEG studies (see for review Boutros et al., 2008) is even more complex due to methodological differences such as beta band having been analyzed as one or more sub-components. It has been postulated that communication within the fronto-parieto-temporal attentional network is characterized by transient long-range phase synchronization in the beta-band (Schnitzler and Gross, 2005); the diffuse alterations seen in LDD (hyperconnectivity in beta1 and hypoconnectivity in beta2) may be different expressions of the same dysfunction in attentional and salience-related networks. Interestingly at molecular level, beta oscillations and coherence have been linked with GABA-B-R1 Gene Polymorphism in healthy individuals (Winterer et al., 2003). Since GABAergic transmission is profoundly impaired in schizophrenia (Schmidt and Mirnics, 2015), future EEG-SFC studies could investigate if beta-band disconnectivity may be considered as an intermediate phenotype of GABAergic dysfunction at system level.

### Gamma band abnormalities

We have found increased EEG-SFC in this band in SDD and decreased in LDD compared with controls. These differences were not only seen in neighboring ROI pairs but also in remote ones, in line with the role of gamma oscillations in long-range synchronization function and dysfunction (Buzsaki and Wang, 2012; Lehmann et al., 2014). A recent study in first episode medicated SCZ using FC methods reported increased gamma activity in left hemisphere sources localized in the infero-orbitofrontal, lateral, and medial temporal and inferior parietal areas. Interestingly, SCZ with low positive and disorganization symptoms showed higher gamma connectivity (Andreou et al., 2014). The dependency of gamma band connectivity patterns on disease duration might be partially explained by differences in inhibitory/excitatory neurotransmission. Recent magnetic resonance spectroscopy studies (Kegeles et al., 2012; Natsubori et al., 2014) have shown increased prefrontal GABA and glutamate in the early stages and a decreased in chronic patients. Moreover, a recent meta-analysis showed greater age-dependent decrease in glutamate and glutamine concentration in schizophrenia compared to controls (Marsman et al., 2013). Our findings have interesting similarities with those from studies of animal models of psychoses that showed increased gamma oscillations after acute administration of ketamine (Pinault, 2008) and decrease after chronic administration (McNally et al., 2013). Recent studies have contributed to better understand the role of gamma oscillations in human brain function (Buzsaki and Wang, 2012) and its implications in schizophrenia (Sun et al., 2011; Uhlhaas, 2011; Uhlhaas and Singer, 2013). In particular, GABAergic interneurons with glutamate regulating effect through N-methyl-D-aspartate (NMDA) receptors have been postulated to be responsible for the dysfunction of gamma oscillations observed in schizophrenia (Spencer, 2009; Gonzalez-Burgos and Lewis, 2012). Finally, as showed in basic (Kocsis et al., 2014), computational (Komek et al., 2012; Wang and Wong-Lin, 2013) and human studies (Demiralp et al., 2007; Komek et al., 2012) a relevant role in the generating and modulating gamma activity, via regulation of GABAergic (Andersson et al., 2012b) and glutamatergic transmission (Andersson et al., 2012a), is also played by dopamine (Herrmann and Demiralp, 2005; Furth et al., 2013), a key neurotransmitter in the pathophysiology of schizophrenia. Due to action of dopamine receptor antagonists in suppressing the gamma activity in humans (Ahveninen et al., 2000) and in animal models (Dejean et al., 2011), we could speculate that prolonged exposition to antipsychotics might account for the decreased gamma connectivity observed in the LDD group.

#### The effect of disease duration on aberrant connectivity of schizophrenia

Long-term effects of schizophrenic disease on brain structure and function have been extensively documented. These include decreased gray matter volume (Glahn et al., 2008), ventricular enlargement (Vita et al., 2006), focal alteration of white matter tracts (Ellison-Wright and Bullmore, 2009) and reduced obligatory auditory cortical responses (Todd et al., 2008). Cognitive abilities as well as affective domains are also affected by disease duration (Fujino et al., 2014). The duration of the schizophrenic illness is strongly associated with long-term treatment with antipsychotic drugs. Recent evidence has shown that exposure to antipsychotic treatment is associated with loss of cortical gray matter (Vita et al., 2012, 2015) and this has generated a debate as to whether the effect of “disease duration” on brain structures and functions in schizophrenia is an expression of a “natural” progression of the illness or a result of a neuro-degenerative effect of antipsychotics. Moreover, early stages of the schizophrenic disease have different clinical and pathophysiological features from those seen in patients with long disease duration (van Os and Kapur, 2009). Furthermore, positive and negative symptoms associated with acute Ketamine administration in HV are associated with hyperconnectivity (Driesen et al., 2013); this finding has strong analogy with functional changes observed in individuals at high risk for schizophrenia and in the early stages of the disease (Anticevic et al., 2015). The glutamatergic-dependent hyperconnectivity appears to be specific for the early phases of schizophrenia and not a feature present in chronic patients.

## Limitations

Some limitations in the current study must be considered. The pharmacological therapy could be a potential confounding factor on connectivity indices of SCZ. Dopamine receptor antagonists can alter FC and network parameters (Achard and Bullmore, 2007). Our patients had been treated with antipsychotics for several years; however, SDD and LDD patients didn't show significant differences in antipsychotic dosage (in chlorpromazine equivalents) making it unlikely for the reported differences in EEG-SFC metrics between the two groups to be attributable to current treatment effects. A further potentially limiting factor could be the relatively low spatial resolution allowed by the number of channels (37) used in our EEG recordings. While there is sufficient evidence to suggest that increased spatial sampling is one of the factors in achieving higher localization accuracy in EEG studies (Liu et al., 2002), the size of cortical patches used in the present study to define ROIs is well within the resolution allowed by the spatial sampling.

## Conclusions

Our study reports for the first time differences in resting-state EEG-SFC functional connectivity within and between brain areas in medicated schizophrenic patients and differences between short duration vs. long duration of the schizophrenic illness. The main findings were a diffuse increase in EEG-SFC in delta and theta bands and a decreased frontal alpha EEG-SFC. Interestingly, low frequency EEG-SFC was more evident in patients with long disease duration while decreased EEG-SFC appeared to be a stable phenomenon throughout the disease course, supporting the idea of functional specificities of the EEG bands (Uhlhaas and Singer, 2010). Lastly, EEG-SFC in the gamma band showed a complex pattern characterized by increase in SDD and decrease in LDD, which might be partially explained by different inhibitory/excitatory patterns of dysfunction in early-stage vs. chronic schizophrenia.

Globally, our findings could be interpreted either as the expression of long-term changes in mechanisms of neural plasticity of brain networks (neurodegeneration hypothesis), or resulting from prolonged exposition to antipsychotics and the associated dysfunctions in inhibitory/excitatory neurotransmission (antipsychotic driven neurodegeneration hypothesis). Current study is not able to discriminate between these two mechanisms and the clinical applicability of these findings has to be determined with prospective studies.

### Conflict of interest statement

The authors declare that the research was conducted in the absence of any commercial or financial relationships that could be construed as a potential conflict of interest.

## Supplementary material

Supplementary Figure 1 **Regions Of Interest (ROIs) based on 6239 voxels of eLORETA inverse matrix**. Label, centroid coordinates (in MNI space) and the voxel number were reported for each left and right hemisphere ROI. In axial view of BrainNet Viewer template 28 ROIs are represented according to the ROI centroid MNI coordinates.

Supplementary Table 1 **Results of four comparisons (HV vs. SCZ; LDD vs. SDD; HV vs. SDD; HV vs. LDD) for all ROI pairs in the six frequency bands are reported**.
