# Supplementary figures and images for: Altered resting-state EEG source functional connectivity in schizophrenia: the effect of illness duration
Source: Front Hum Neurosci. 2015 May 5;9:234. doi: 10.3389/fnhum.2015.00234 (PMC4419718; doi:10.3389/fnhum.2015.00234)

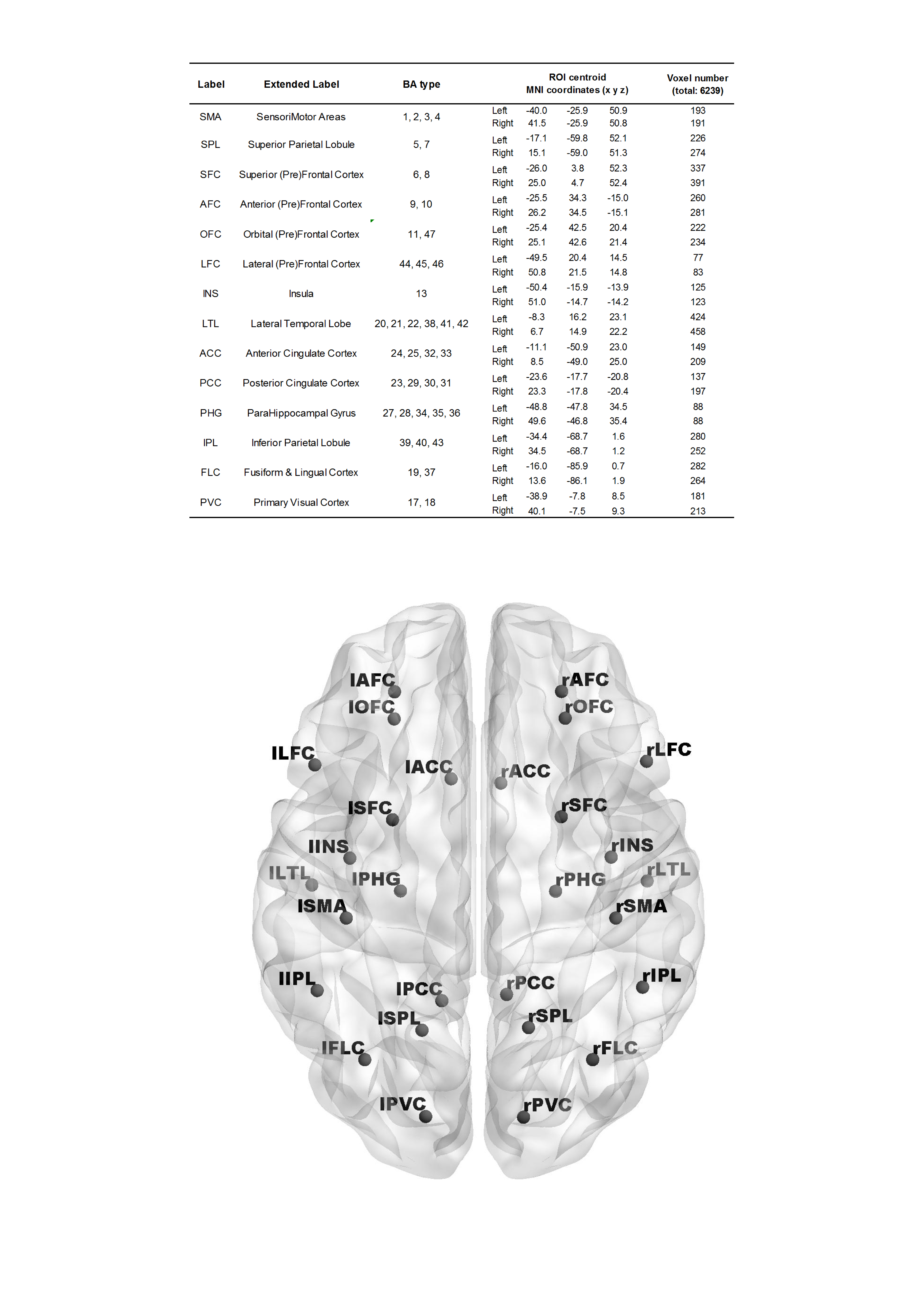

Supplement: Supplementary Figure 1 — Regions Of Interest (ROIs) based on 6239 voxels of eLORETA inverse matrix. Label, centroid coordinates (in MNI space) and the voxel number were reported for each left and right hemisphere ROI. In axial view of BrainNet Viewer template 28 ROIs are represented according to the ROI centroid MNI coordinates. [file Image1.TIFF]
